# Analysis of predictors of malnutrition in adult hospitalized patients: social determinants and food security

**DOI:** 10.3389/fnut.2023.1149579

**Published:** 2023-05-09

**Authors:** Krystel Ouaijan, Nahla Hwalla, Ngianga-Bakwin Kandala, Joelle Abi Kharma, Emmanuel Kabengele Mpinga

**Affiliations:** ^1^Department of Clinical Nutrition, Saint George Hospital University Medical Center, Beirut, Lebanon; ^2^Institute of Global Health, University of Geneva, Geneva, Switzerland; ^3^Department of Nutrition and Food Sciences, American University of Beirut, Beirut, Lebanon; ^4^Department of Epidemiology and Biostatistics, Schulich School of Medicine and Dentistry, Western University, London, ON, Canada; ^5^Division of Epidemiology and Biostatistics, School of Public Health, University of the Witwatersrand, Johannesburg, South Africa; ^6^Faculty of Arts and Sciences, Lebanese American University, Beirut, Lebanon

**Keywords:** social determinants, food security, hospital malnutrition, Global Leadership Initiative on Malnutrition GLIM, prevalence, health coverage, Right to Health

## Abstract

**Background:**

Malnutrition in hospitalized patients is becoming a priority during the patient care process due to its implications for worsening health outcomes. It can be the result of numerous social factors beyond clinical ones. This study aimed to evaluate the link between these various risk factors considered social determinants of health, food security levels, and malnutrition and to identify potential predictors.

**Methods:**

A cross-sectional observational study was conducted on a random sample of adult patients in five different hospitals in Lebanon. Malnutrition was assessed using the Global Leadership Initiative on Malnutrition (GLIM) criteria. Patients were interviewed to collect social and economic characteristics and were categorized into four criteria: (1) area of residence (urbanization level), (2) level of education, (3) employment status, and (4) source of health coverage. The food security level was screened by a validated two-question tool, adapted from the US Department of Agriculture Household Food Security Survey, targeting both quantity and quality.

**Results:**

In a random sample of 343 patients, the prevalence of malnutrition according to the GLIM criteria was 35.6%. Patients with low levels of food security, mainly low quality of food, had higher odds of being malnourished (OR = 2.93). Unemployed or retired patients and those who have only completed only elementary school had higher odds of being diagnosed with malnutrition as compared to those who were employed or had university degrees, respectively (OR = 4.11 and OR = 2.33, respectively). Employment status, education level, and type of health coverage were identified as predictors of malnutrition in the multiple regression model. Household location (urban vs. rural) was not associated with malnutrition.

**Conclusion:**

The social determinants of health identified in our study, mainly the level of education and income level, in addition to food security, were identified as predictors of malnutrition in hospitalized patients. These findings should guide healthcare professionals and national policies to adopt a broader perspective in targeting malnutrition by including social determinants in their nutrition care.

## 1. Introduction

Malnutrition in hospitalized patients has been associated with an increasing rate of complications and worsening outcomes (1). Malnutrition impairs many physiologic functions of the body, impairing the immune system, delaying wound healing, and leading to loss of muscle mass and strength (2). Major consequences resulting from these implications include increased morbidity, increased length of stay, nosocomial infections, and hospital readmission (1, 3). Patients diagnosed with malnutrition have in addition 5-fold higher mortality rate than patients with normal nutrition status (4). Malnutrition among hospitalized patients is typically categorized as disease-related malnutrition, as it is assumed to be mainly caused by the patient's clinical condition and the inflammatory process associated with their current illness (5–7).

However, malnutrition in hospitals may also arise from a combination of factors that extend beyond clinical factors, as observed in community settings (8, 9). An analysis of data from the Healthcare Cost and Utilization Project (HCUP) in the United States revealed a correlation between patients' income levels and their nutritional status upon admission to the hospital, with a higher incidence of malnutrition diagnosed in patients below the 50th percentile of income (10). These results highlight that a person's socioeconomic status can significantly affect their health, including their nutritional wellbeing (11). The World Health Organization (WHO) has long established that various factors, such as education level, employment, and urbanization, in addition to income, play a role in shaping population health via different mechanisms and have been categorized as social determinants of health (12, 13). However, studies on the impact of these determinants on nutritional status have been scarce and focused only on the growth of children (14, 15). More specifically, the influence of these determinants on the nutritional status of adult hospitalized patients has not been accounted for in previous studies.

Food security, another significant social determinant, has also an impact on both the quantity and quality of food intake affecting, as a result, the nutritional status of the hospitalized patient (16). Decreased food intake caused by insufficient food quantity is a primary contributor to weight loss, while inadequate food quality leads to reduced intake of essential nutrients and impacts nutritional status in patients (2). Although studies on food security have mainly examined the association between poor nutrient-dense foods and obesity, there is still limited evidence linking food security with malnutrition in healthcare settings, particularly among adults (17). Data mainly focus on growth decline in children, and research on adults in healthcare settings is scarce (16).

The social determinants of health along with food security are taken into consideration as part of the Right to Health, which dictates their availability and equitable accessibility (18). The Right to Health is recognized as a fundamental part of Human Rights in all international treaties (11). The essential elements of the Right to Health under the Human Rights approach ensure that all people have equal access to the underlying determinants of good health (18). Understanding the relationship between social and economic factors with the risk of malnutrition in hospitalized patients adds an important perspective of strategies targeting the patient's Right to Health (19).

Lebanon is a small country in the Middle East Region that is divided into five main districts with an estimated population of 6,847,712 and 144 hospitals comprising 11,742 beds (20, 21). Studies on the prevalence of malnutrition in hospitalized patients have been modest with a small study reporting a rate of 37.4% in one hospital (22). The country has recently witnessed a severe financial crisis. According to the World Bank, a drop of 36.5% in gross domestic product per capita has reclassified the country as a lower-middle-income country instead of an upper-middle-income country (21). These drastic changes have directly affected employment status impacting household incomes and therefore food security and the extent of healthcare coverage. The aim of this study was to assess the association between indicators of social determinants of health and food security with malnutrition in adult hospitalized patients. We also aimed to determine whether any of these factors are potential predictors of nutritional status. The results of this study would suggest taking a social perspective when identifying malnutrition in hospitalized patients and providing guidance for national policies on including malnutrition in hospitalized patients under the Right to Health framework.

## 2. Materials and methods

### 2.1. Study population

Patients were enrolled as a part of a cross-sectional, observational, and multicenter study intended to assess the national prevalence of malnutrition from May to October 2021. They signed an informed consent form after being introduced to the aim and process of the study. A total of five hospitals, one hospital from each of the five districts of Lebanon, were selected by convenience sampling. All adult patients, men and women aged 18 years and above, admitted to the different wards of the hospitals during the period of data collection were recruited within 48 h of admission. Patients with dementia or other cognitive impairment were also included, and the caregivers were approached to sign the consent form and fill the part of the questionnaire. Exclusion criteria included the following wards: gynecology, intensive care unit, psychiatry, and short stay of <48 h because of the inability of conducting questionnaires.

### 2.2. Data collection and social determinants

The patient's basic characteristics, including age, gender, marital status, and admission diagnosis, were recorded. The World Health Organization and Office of Disease Prevention and Health Promotion identify various indicators as integral to social determinants of health impacting directly health outcomes in their Healthy Report 2030 (13, 23). Four of these indicators were considered, and patients were interviewed accordingly: (1) area of residence (urbanization level), (2) level of education, (3) employment status, and (4) source of health coverage (11, 12). The source of health coverage that applies to the country context includes National Social Security Fund (NSSF), private insurance, and financial aid from governmental and non-governmental organizations.

### 2.3. Food security

The level of food insecurity was screened using a simplified tool based on two questions adapted from the 2000 United States Department of Agriculture Report on Food Security Measurement Project (24). It was demonstrated that a two-item screening tool has high sensitivity and specificity and is a practical tool for use in surveys conducted in healthcare settings (25–27). The two questions (Q1 and Q4) from the Food Security Scale were selected to focus on the patient's perception of food availability in the household. The first question was “Which of these statements best describes the food eaten in the household in the last 12 months?” The response categories were as follows:

1. enough of the kinds of food we want to eat
2. enough but not always the kinds of food we want
3. sometimes not enough to eat
4. often not enough to eat.

The second question was “Which of these statements best describes the quality of food eaten in the household in the last 12 months?” The response categories were determined based on the patient's description of the number of food groups they consume as follows (24):

1. very good
2. good
3. average
4. poor.

### 2.4. Nutritional status

The Global Leadership Initiative on Malnutrition (GLIM) was used to diagnose malnutrition and its severity in hospitalized patients (5). It is a two-step process by first identifying at least one phenotypic criterion and one etiologic criterion and second assessing the severity of malnutrition as “moderate” and “severe” based on the phenotypic criterion. Anthropometrics, including height, weight, body mass index (BMI), and mid-upper arm muscle circumference (MUAC), were used to evaluate the phenotypic criteria. Patients were interviewed for the history of weight loss, appetite, and record of food intake. Food intake was assessed using the dietary recall of meals consumed before hospital admission and categorized as <50% of estimated needs in >1 week or any reduction for >2 weeks. C-reactive protein levels (CRPs) were retrieved from the available blood tests from the patient's records. Reduced food intake retrieved from the patient's interviews and inflammatory condition assessed by their CRP levels retrieved from the patient's files was the etiologic criteria. Cutoff points of the different etiologic and phenotypic criteria are described in Table 1.

**Table 1:** Global Leadership Initiative on Malnutrition GLIM criteria for the diagnosis of malnutrition (43).

**Phenotypic criteria**			**Etiologic criteria**	
	**Severity level**	**Moderate**	**Severe**	
Weight loss	>5–10% within past 6 months or 10-20% beyond 6 months	>10% within past 6 months, or >20% beyond 6 months	Reduced food intake	<50% of Estimated Needs in > 1 week or any reduction for >2 weeks
Low BMI	<20 if <70 years, >22 if >70 years	<18.5 if <70 years, <20 if <70 years		any chronic GI condition that adversely impacts food assimilation or absorption
Reduced muscle mass	MUAC^a^ < 23	MUAC < 20	Inflammation	Elevated C-Reactive Protein (CRP) levels

a Mid-Upper Arm Muscle Circumference.

### 2.5. Statistical analysis

Statistical analysis was performed using STATA v17.1. Descriptive analysis was used to summarize the study variables and to check for out-of-range values. Continuous variables were described using mean and standard deviations, while frequencies and percentages were used to represent categorical variables. Shapiro–Wilk was used to assess data normality. The median and interquartile range (IQR) were used to describe the non-parametric variables. A series of simple logistic regressions were conducted at the bivariate level to identify potential predictors of malnutrition. A multiple logistic regression model was run thereafter to assess the independent associations between malnutrition status and patients' social determinants and food security level. Variables were selected for inclusion in the model based on a *p*-value of < 0.2 at the bivariate level. All reported *p*-values were evaluated at a significance level of 5%.

## 3. Results

### 3.1. Sociodemographic characteristics

A total of 343 participants were enrolled in this study from May to October 2021. Demographics and social characteristics are presented in Table 2. The mean age was 60 years (SD: 17 years), and the majority of the patients were <70 years old (65.89%). Almost half of the patients were male (54.81%), and the majority were married (70.55%). The majority of households (62.10%) were located in urban areas. In total, 27.99% of participants had university degrees, but more than half were not working (58.6%).

**Table 2:** Sociodemographic characteristics of patients (*N* = 343).

	***N* (%)**
**Age**	
<70 years old	226 (65.89%)
≥70 years old	117 (34.11%)
**Gender**	
Male	188 (54.81%)
Female	155 (45.19%)
**Marital status**	
Single	45 (13.12%)
Married	242 (70.55%)
Divorced	17 (4.96%)
Widowed	39 (11.37%)
**Level of education**	
No schooling	32 (9.33%)
Primary school	59 (17.20%)
Intermediate school	66 (19.24%)
High school	64 (18.66%)
Technical diploma	26 (7.58%)
University degree	96 (27.99%)
**Work status**	
Not working	157 (45.77%)
Employee full time	91 (26.53%)
Employee part time	11 (3.21%)
Self-employed	29 (8.45%)
Retired	55 (16.03%)
**Household location**	
Urban	213 (62.10%)
Countryside	130 (37.90%)
**Health coverage**	
None	30 (8.75%)
NSSF^a^	86 (25.07%)
Private insurance	84 (24.49%)
Combination of NSSF1 and insurance	40 (11.66%)
Army or other governmental institution	82 (23.91%)
Non-governmental organization	21 (6.12%)

a National social security fund.

### 3.2. Nutritional status of patients

Using the GLIM diagnostic criteria, a total of 35.57% of patients (*n* = 122) were identified as malnourished, 21.28% (*n* = 73) had a moderate level of malnutrition, and 14.29% (*n* = 49) were classified as being severely malnourished. An equal proportion (50%) of malnourished patients were distributed in male and female populations. Among the 122 patients identified as malnourished, the most dominant phenotypic criterion was “weight loss” accounting for 76.7% followed by low muscle mass (57.5%) and low BMI (31.2%). Decreased food intake was the most common etiologic criterion identified (88%) followed by inflammatory status (60.7%).

### 3.3. Level of food security

Referring to the quantity of food consumed in the first question, the majority of the patients (58.31%) described their household food to be “enough but not always the kinds of food we want” as shown in Table 3. Only six patients (1.75%) responded as not having enough food to eat. When referring to the quality of food in the household in the second question, responses were mainly distributed between two categories: good (32.36%) and average (38.19%).

**Table 3:** Distribution of level of food security among patients (*N* = 343).

	**N (%)**
**Food security (quantity)**	
Enough of the kinds of food we want to eat	87 (25.36%)
Enough but not always the kinds of food	200 (58.31%)
Sometimes not enough to eat	50 (14.58%)
Often not enough to eat	6 (1.75%)
**Food security (quality)**	
Very good	80 (23.32%)
Good	111 (32.36%)
Average	131 (38.19%)
Poor	21 (6.12%)

### 3.4. Association of malnutrition with social determinants

Table 4 describes the bivariate associations between malnutrition and different sociodemographic characteristics. The odds of being malnourished according to the GLIM criteria were higher among patients of older age (≥70 years old, *p* < 0.001) compared to those of younger age. Gender and marital status were not significantly associated. As for the four indicators identified as social determinants, unemployed or retired patients (*p* < .001) and those who had completed basic schooling (*p* = 0.004) or no schooling at all (*p* = 0.047) had higher odds of being malnourished as compared to those employed or had university degrees, respectively. Household location (urban vs. rural) and type of health coverage were not significantly associated with being malnourished.

**Table 4:** Bivariate associations between diagnosis of malnutrition and social determinants and level of food security.

	**Odds ratio (OR)**	**CI**	***P*-value**
**Age** ^a^			
≥70 years old	4.16	2.58; 6.70	<0.001^**^
**Gender** ^b^			
Female	1.35	0.86; 2.10	0.184
**Employment status** ^c^			
Not working	4.11	2.43; 6.95	<0.001^**^
**Level of education** ^d^			
No schooling	2.33	1.01; 5.39	0.047^*^
Primary school/intermediate school	2.36	1.32; 4.21	0.004^*^
High school/technical diploma	1.43	0.75; 2.70	0.276
**Marital status** ^e^			
Married	1.19	0.73; 1.96	0.470
**Household location** ^f^			
Countryside	0.88	0.56; 1.39	0.603
**Health coverage** ^g^			
NSSF	1.03	0.44; 2.40	0.947
Private insurance	0.5	0.21; 1.20	0.123
Combination of NSSF and insurance	0.57	0.21; 1.55	0.273
Army or other governmental institution	1.17	0.50; 2.74	0.712
Non-governmental organization	0.75	0.23; 2.40	0.628
**Food Security (Quantity)** ^h^			
Enough of the kinds of food we want to eat	0.88	0.52; 1.49	0.650
Enough but not always the kinds of food sometimes/often not enough to eat	1.11	0.56; 2.21	0.763
**Food security (quality)** ^i^			
Good	1.24	0.67; 2.28	0.491
Average	1.15	0.64; 2.08	0.643
Poor	2.93	1.09; 7.85	0.032^*^

a reference group “ <70 years old”,

b reference group “males”,

c reference group “working”,

d reference group “university degree”,

e reference group “not married”,

f reference group “urban”,

g reference group “none”,

h reference group “Enough of the kinds of food we want to eat”,

i reference group “very good”

* *p* < 0.05.

### 3.5. Association of malnutrition with the level of food security

Patients who described in the first question the quality of the food eaten to be “poor” compared to “very good” have higher odds of being malnourished (*p* = 0.032). There was no association between malnutrition and the reported description of food quantity in the second question (*p* = 0.4234) as shown in Table 4.

### 3.6. Multiple logistic regression and potential predictors of malnutrition

Age, work status, district, and type of health coverage were found to be independent predictors of malnutrition diagnosis as shown in Table 5. Specifically, patients of older age (≥70 years old, *p* < 0.001) and unemployed/retired (*p* < 0.001) had higher odds of being diagnosed with malnutrition compared to their counterparts. As for food security, patients who described the quality of the food eaten to be “poor” compared to “very good” in the first question had higher odds of being malnourished (*p* = 0.066), but the results were borderline significant. However, patients who had private insurance as medical coverage means had lower odds of being diagnosed with malnutrition (*p* = 0.033). The Hosmer and Lemeshow goodness-of-fit test indicates that our model fits the data well with *p*-values of 0.7247.

**Table 5:** Adjusted multiple logistics regression model of diagnosis of malnutrition and social determinants and level of food security.

	**Odds ratio (OR)**	**95% CI for OR**	***P*-value**
**Age** ^a^			
≥70 years old	3.03	1.55; 5.90	0.001^**^
**Gender** ^b^			
Female	0.88	0.49; 1.57	0.684
**Employment status** ^c^			
Not working	2.50	1.23; 5.09	0.011^**^
**Level of education** ^d^			
No schooling	1.53	0.46; 5.09	0.481
Primary school/intermediate school	1.10	0.51; 2.42	0.799
High school/technical diploma	0.75	0.35; 1.64	0.473
**Health coverage** ^e^			
NSSF	0.85	0.31; 2.36	0.759
Private insurance	0.30	0.10; 0.90	0.033^*^
Combination of NSSF and insurance	0.32	0.09; 1.13	0.079
Army or other governmental institution	0.84	0.29; 2.42	0.750
Non-governmental organization	0.69	0.15; 3.19	0.645
**Food security (quality)** ^f^			
Good	1.75	0.82; 3.73	0.151
Average	1.48	0.66; 3.35	0.343
Poor	3.54	0.92; 13.61	0.066

a reference group “ <70 years old”,

b reference group “males”,

c reference group “working”,

d reference group “university degree”,

e reference group “none”,

f reference group “very good”

* *p* < 0.05.

## 4. Discussion

The nutritional status of hospitalized patients in this study was assessed and diagnosed using the GLIM criteria. It is a newly proposed diagnostic tool based on a global set of criteria that take into consideration different characteristics of malnutrition, including weight loss, muscle mass, and food intake (28). It is considered an evolving concept that was designed to provide a more specific diagnosis of malnutrition and has been validated in numerous studies (28–31). The prevalence rate of malnutrition among hospitalized patients in this study was found to be 36.7% using the GLIM criteria. In an international multicenter study that included two hospitals in Lebanon and was conducted in 2008, nutrition screening was done using Nutrition Risk Screening (NRS) and reported a lower rate of 22% of patients being at risk of malnutrition (32). Although both studies were done on adult hospitalized patients without excluding any medical conditions, they differ in two major criteria. First, the study used a screening tool as compared to the use of a diagnostic tool in our study. In addition, it was carried out in only one district of Lebanon including 273 patients as compared to our study that was carried out in all five districts including 343 patients. However, a notable increase in the prevalence from 22% of patients at risk to 36.7% of patients diagnosed with malnutrition is observed. A possible explanation for this increase is the drop in GDP that the country has experienced leading to a drastic financial crisis (21).

This proposed explanation further supports our hypothesis that malnutrition in hospitalized patients is influenced not only by well-known medical and clinical conditions but also by social and economic factors. As a matter of fact, a financial crisis will affect the ability to purchase enough food of good quality affecting in return the nutritional status of the patients (33). In our study, the risk of food insecurity was screened using a valid adapted tool focusing on both the quantity and quality of food (34). Nearly 60% of patients reported that their food intake was sufficient in quantity but inadequate in variety, as they lacked access to different types of food groups. This lack of adequacy described by the patients in our study was significantly associated with malnutrition despite the food quantity. Other numerous studies have always focused on exploring food insecurity either starvation in the community as a consequence of unavailability of food or obesity as a consequence of unhealthy food choices (16, 35). In our study, we used a regression model and identified the level of food security as a predictor of malnutrition in hospitalized adults. Patients who had a poor level of food security identified by the adapted tool we used had higher odds of 3.56 to being malnourished as compared to patients categorized with a good level.

Food security is recognized as a component of the social determinants of health that include education, economic stability, and access to healthcare (36). These fundamental determinants have been linked to adverse health outcomes and are considered key drivers of health equity. Research has primarily concentrated on the pediatric population in the community and has established a correlation between low income and education levels with child stunting as an indicator of poor nutritional status (37, 38). In our study population in the hospital setting, employment status and education level were highly associated with malnutrition. Patients who were not working or had completed only elementary school had higher odds of being diagnosed with malnutrition. In addition, employment status was considered a predictor of malnutrition in hospitalized patients in our regression model (OR = 4.1). Malnutrition in older people living in the community was also associated with low educational levels in a recent systematic review (39). On the other hand, marital status was not associated with the level of malnutrition in our population and cannot be determined as a risk factor.

Another predictor of malnutrition in our study was the type of health coverage. Patients who had been insured in private insurance had significantly lower odds of being malnourished as compared with patients with no health coverage or relying on social security funds and non-governmental aid. Private insurance in Lebanon is prohibitively expensive and typically only obtained by individuals from higher socioeconomic groups reflecting a correlation between income level and risk of malnutrition. This correlation has also been demonstrated when studying the nutritional status of children and older adults in the community (33, 39). The type of residence area, being urban or rural, was not associated with malnutrition in our model. The small surface area of Lebanon (10,452 km^2^) has decreased the differences in the level of urbanization between the cities and rural areas, and therefore, a discrepancy could not be identified.

The association that we have demonstrated between social determinants and food security should alarm healthcare professionals to broaden their perspective when identifying malnutrition in hospitalized patients. When conducting nutritional assessments, including the GLIM criteria or any validated tool, it is advisable to incorporate a social dimension and identify any factors that increase the risk of malnutrition, such as food insecurity, low income, or low literacy levels (29, 40). When developing a management plan for malnutrition in hospitalized patients, it is crucial to address social determinants and food security as essential components. Healthcare professionals are used to focusing primarily on biomedical and clinical care that has been recently described as a downstream approach aiming to treat symptoms of malnutrition without targeting root causes (41, 42). Healthy People 2030 initiative has recently proposed a more proactive approach that targets the causes of diseases at a macro-level. This initiative acknowledges the economic and social factors that are typically beyond the patient's control (36). In order to provide effective nutritional care, healthcare professionals should review the patient's living and working conditions and address the social determinants of health directly (33). Through this tailored approach, healthcare professionals can prioritize enhancing food security, education, and income levels, even for hospitalized patients, as a means of achieving the Right to Health at a broader national level (18).

This study has several strengths. First, it has a heterogeneous population because it included patients from five hospitals across different areas and admitted to various wards. Second, the identification of malnutrition was not done by a screening process but was determined through a systematic nutritional assessment using the new GLIM criteria. Third, it was the first study to our knowledge to investigate the association of social determinants with malnutrition measured in hospitalized patients and to identify potential predictors. This study also has some limitations. First, social and economic indicators were collected from the patients and their caregivers through a questionnaire and they had some reservations while answering the questions. Second, a direct question on income level could not be collected due to the severe devaluation of the national currency and the inadequacy of any relevant categorization. Third, food security was only addressed at a screening level using a two-item questionnaire as hospitalized patients were less responsive to surveys of longer duration.

## 5. Conclusion

To conclude, our study found a malnutrition prevalence rate of 35.57% in hospitalized patients in Lebanon. We also identified social determinants of health, including education level, income level, employment status, and health coverage, as factors associated with malnutrition, along with food security. These determinants were also recognized as predictors of malnutrition in hospitalized patients. Our findings suggest that healthcare professionals should consider adopting a broader perspective in targeting malnutrition in their patients. Their approach should aim to address the underlying causes of malnutrition beyond clinical factors by incorporating social determinants into their nutritional care assessments. National authorities should also prioritize addressing the social determinants of health in their policy agenda to improve malnutrition at the clinical level.

## Data availability statement

The original contributions presented in the study are included in the article/supplementary material, further inquiries can be directed to the corresponding author.

## Ethics statement

The studies involving human participants were reviewed and approved by Institutional Review Board of the American University of Beirut. The patients/participants provided their written informed consent to participate in this study.

## Author contributions

EK, KO, NH, and N-BK: conceptualization and methodology. KO: data collection and writing—original draft preparation. KO and JA: formal analysis. EK, NH, and N-BK: writing—review and editing. EK: supervision. KO and NH: funding acquisition. All authors contributed to the article and approved the submitted version.
